# Supplementary material for: Predicting visual function by interpreting a neuronal wiring diagram
Source: Nature. 2024 Oct 2;634(8032):113–23. doi: 10.1038/s41586-024-07953-5 (PMC11446822; doi:10.1038/s41586-024-07953-5)
Supplement: Supplementary file 6 — CRF and ERF predictions for individual TmY4 and TmY9 cells. Analogous to Supplementary Data 3, but for TmY target types. Shown are the top four monosynaptic pathways, the strongest pathway passing through each of the top ten intermediary types (ranking from Extended Data Fig. 7), and the trisynaptic pathway Tm1–TmY–Dm3–TmY (see the section entitled Prediction of spatial normalization). [file 41586_2024_7953_MOESM6_ESM.zip › DataS4/TmY9qΓèÑ/720575940635007513.pdf]

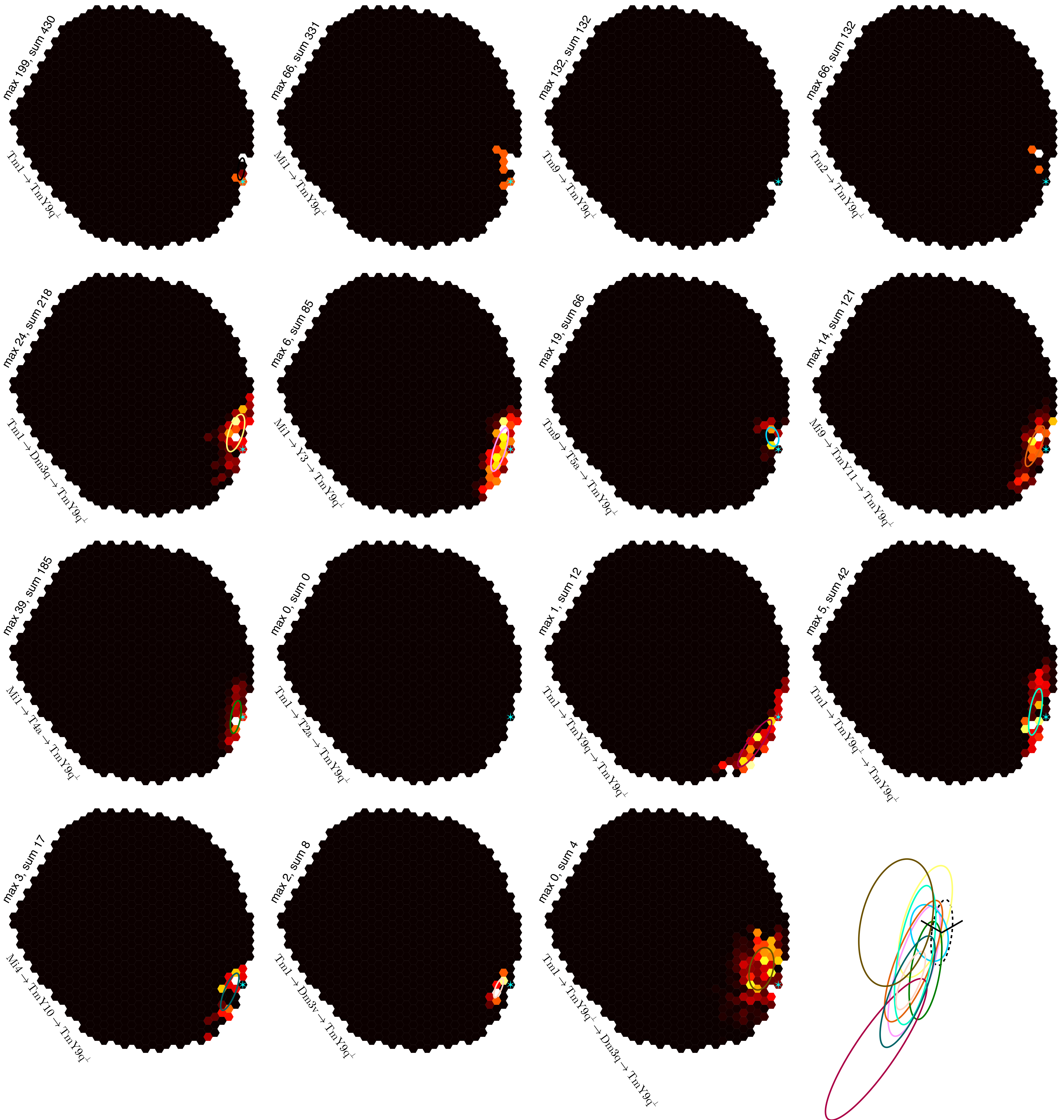
max 199, sum 430
Tm1 → TmY9q⊥
max 66, sum 331
Mi1 → TmY9q⊥
max 132, sum 132
Tm9 → TmY9q⊥
max 66, sum 132
Tm2 → TmY9q⊥
max 24, sum 218
Tm1 → Dm3q → TmY9q⊥
max 6, sum 85
Mi1 → Y3 → TmY9q⊥
max 19, sum 66
Tm9 → T5a → TmY9q⊥
max 14, sum 121
Mi9 → TmY11 → TmY9q⊥
max 39, sum 185
Mi1 → T4a → TmY9q⊥
max 0, sum 0
Tm1 → T2a → TmY9q⊥
max 1, sum 12
Tm1 → TmY9q → TmY9q⊥
max 5, sum 42
Tm1 → TmY9q⊥ → TmY9q⊥
max 3, sum 17
Mi4 → TmY10 → TmY9q⊥
max 2, sum 8
Tm1 → Dm3v → TmY9q⊥
max 0, sum 4
Tm1 → TmY9q⊥ → Dm3q → TmY9q⊥
